# Artificial intelligence models for point-of-care ultrasound diagnostics in dogs

**DOI:** 10.3389/fvets.2026.1729114

**Published:** 2026-03-10

**Authors:** Ricardo Martinez, Krysta Lynn Amezcua, Sofia I. Hernandez Torres, Theodore Winter, Igor Yankin, Emilee Venn, Eric J. Snider, Thomas Edwards

**Affiliations:** 1 Texas A&M College of Veterinary Medicine & Biomedical Sciences, College Station, TX, United States; 2 US Army Institute of Surgical Research, Joint Base San Antonio-Fort Sam Houston, San Antonio, TX, United States; 3 Department of Surgery, Long School Medicine, UT Health San Antonio, San Antonio, TX, United States

**Keywords:** artificial intelligence, effusion, pneumothorax, point-of-care diagnostics, trauma, ultrasound

## Abstract

**Introduction:**

Point-of-care ultrasound (POCUS) for the purpose of Focused Assessment with Sonography for Trauma (FAST) is an essential diagnostic tool for triage in canine patients, but its accuracy is highly operator-dependent. Artificial intelligence (AI) offers a potential solution for improving diagnostic capability by providing real-time, automated interpretation of ultrasound images, particularly in resource-limited or pre-hospital settings. This study evaluated the feasibility and diagnostic performance of deep learning models for detecting life-threatening effusions and pneumothorax (PTX) in dogs.

**Methods:**

Five healthy military working dogs (MWDs) and twenty client-owned dogs (22–55 kg) were prospectively enrolled. MWDs were negative for injury for baseline data capture. Client-owned dogs with confirmed abdominal, pleural, pericardial effusion, or PTX were imaged using POCUS. Ultrasound clips were reviewed for quality, curated by experts, converted to image frames from videos, and used to train, optimize, and evaluate different convolutional neural network (CNN) architectures at all FAST scan sites.

**Results:**

Models were developed for each scan site with varied performance. Diaphragmatico-hepatic scan site models achieved excellent performance (recall 98%, accuracy 97%) while the pericardial models (recall 87%, accuracy 85%) and chest tube site models (recall 81%, accuracy 88%) demonstrated good performance. The spleno-renal/hepato-renal models (recall 83%, accuracy 78%) and cysto-colic models (recall 84%, accuracy 77%) achieved fair performance. Model prediction overlays confirmed that the models for each site focused on clinically relevant regions during predictions.

**Discussion:**

Deep learning models can accurately detect effusion and PTX in canines using POCUS, with variable performance at individual sites. Limitations included small sample sizes, inclusion of only blunt trauma and non-traumatic pathology, class imbalances, and variability in the volume and location of effusion on presentation. Expanding the training datasets and refining pre-training strategies may enhance performance. These findings support the feasibility of AI-assisted ultrasound to augment triage and pre-hospital decision-making in veterinary emergency care.

## Introduction

Point-of-care ultrasound (POCUS) has emerged as a critical diagnostic tool in veterinary emergency and critical care, particularly in the triage of trauma patients. POCUS protocols encompassing abdominal and thoracic evaluations have been validated in canine trauma for the detection of abdominal effusion, pleural effusion, pericardial effusion, and pneumothorax (PTX) (1–4). These standardized approaches have demonstrated value in triage, monitoring, and prognostication (5, 6). However, interpretation remains highly operator-dependent and subject to variable expertise, particularly in resource-limited environments.

Despite the importance of POCUS in early recognition of hemorrhage and life-threatening conditions, limitations exist in its widespread application. Interpretation requires significant training and practice, and access to highly skilled personnel may be delayed or unavailable in remote or combat settings. This presents a critical gap in current triage capability for military working dogs (MWD), where timely intervention can be the difference between survival and mortality (7–9). Furthermore, even under optimal conditions, diagnostic accuracy of eFAST (extended Focused Assessment with Sonography for Trauma) protocols in both human and veterinary applications remains variable (10). A review of 75 studies representing over 24,000 patients reported pooled sensitivities and specificities for detection of PTX (69 and 99%), pericardial effusion (91 and 94%), and intraperitoneal free fluid (74 and 98%) (11). Staub et al. (12) further reported a sensitivity of 81% and specificity of 98% for ultrasound detection of PTX, highlighting strong performance in some contexts but persistent gaps in sensitivity. Similarly, Boysen (1) reported that when performed by physicians with extensive experience and surgical and emergency residents with minimal training, FAST protocols demonstrated good sensitivity (81%−98%) and specificity (98%−100%) for detection of free fluid in body cavities. By contrast, a review of prehospital extended FAST or eFAST performed by helicopter emergency medical services clinicians in 411 trauma patients demonstrated markedly lower sensitivity: 25% for intraperitoneal fluid, 38% for PTX, and only 17% for hemothorax and pericardial effusion, though specificity remained above 95% for all categories (13). Smith et al. (14) also reported that FAST for intra-abdominal injury achieved a sensitivity of 56%, specificity of 98%, and overall accuracy of 89%. Collectively, these human studies demonstrate that while specificity is consistently high, sensitivity is often suboptimal, with performance heavily influenced by operator experience and practice environment.

In a study of 145 client-owned dogs evaluated within 24 h of injury, an overall sensitivity of 78% and specificity of 93% was reported for PTX detection, with sensitivity ranging from 45 to 95% and specificity ranging from 91 to 95% depending on operator experience when comparing results to thoracic radiography (2). Furthermore, in a prospective study of 13 dogs and two cats with sustained blunt force and penetrating trauma, ER clinicians, house officers, and radiology house offers of varied experience levels evaluated the presence of PTX with an overall accuracy of 53%, with sensitivity ranging from 0 to 17% and specificity ranging from 78 to 83% when compared to gold standard CT findings (15). In the same study, peritoneal effusion was identified with an overall accuracy of 80%−93%, sensitivity of 75%, and specificity of 82%−100%. Finally, a prospective observational study on the use of POCUS in 64 trauma affected dogs demonstrated positive agreement of 93 and 94% for PTX and abdominal effusion, respectively, when compared with centesis findings, and negative agreement of 94 and 100%, respectively (16). Therefore, in veterinarian studies, PTX detection was reported with 53% accuracy, 0%−95% sensitivity, and 78%−83% specificity. Furthermore, the identification of abdominal effusion showed accuracies ranging from 80 to 93%, sensitivities of 75%−94%, and specificities of 82%−100%. Accuracies varied depending on operator experience, cause of injury (blunt vs. penetrating trauma), and method of injury verification across published studies.

Artificial intelligence (AI) offers a potential solution to address gaps in triage capability by providing real-time, automated interpretation of ultrasound images when highly trained personnel are unavailable. AI has been increasingly applied in veterinary diagnostic imaging, demonstrating promising accuracy for radiographic and ultrasonographic interpretation (17–21). A prior study has shown that deep learning models can classify thoracic radiographs in small animals with accuracy comparable to veterinarians and can augment clinical decision-making by reducing error rates in image interpretation (22). Recently, AI-assisted approaches have also been explored in veterinary ultrasound by our research team, including work focused on military working dogs, highlighting the feasibility of automated POCUS interpretation in high-stakes environments (23).

In human medicine, AI-based systems for ultrasonography have demonstrated utility in emergency medicine and critical care, supporting clinicians in the detection of effusion and PTX (24–27). Translating these advances to veterinary medicine, particularly in the context of MWD triage, represents a novel and clinically meaningful step forward. Importantly, establishing performance benchmarks for AI based ultrasound interpretation is necessary to ensure clinical utility.

Given that prior eFAST studies demonstrate limited sensitivity despite high specificity, this study targets an AI model performance threshold of ≥80% recall and ≥75% accuracy, which was determine by the authors to align with reported performance metrics in the literature and would point toward the generation of clinically relevant needs AI models with a limited dataset. The present study addresses the need for emergency diagnostic support for military working dogs through the development of AI models for the detection of pleural effusion, abdominal effusion, pericardial effusion, and PTX through standardized eFAST ultrasound (US) imaging protocols.

## Materials and methods

### Regulatory approvals

Subject data for this study was acquired from two sources. Images from MWDs undergoing either orchiectomy or abdominal ultrasound with no abnormal effusions or PTX were collected and used to train the AI using normal images. Data collection from healthy MWDs was approved by the Department of Defense Military Working Dog Veterinary Services Institutional Animal Care and Use Committee with appropriate second level review. Data collection from dogs with naturally occurring effusions and PTX was conducted at Texas A&M University Veterinary Medical Teaching Hospital under the Texas A&M IACUC with appropriate second level review. Client-owned canine patients were prospectively enrolled between May 2024 and August 2025 for ultrasound image collection. Enrollment was limited to patients presenting to the Texas A&M University Small Animal Hospital with abdominal effusion, pleural effusion, pericardial effusion, PTX, or any combination of these conditions identified through POCUS during hospitalization. Written client consent was obtained prior to image collection. Because the target population for this study was MWDs, inclusion criteria required a body weight between 22 and 55 kg and age of 1–14 years. While the target population was patients with trauma, any non-traumatic causes of effusions or PTX were also included to provide a larger learning set given ultrasonographic similarities of pathology. Brachycephalic breeds (e.g. English bulldogs, boxers, bullmastiffs) were excluded from enrollment given significance in thoracic anatomic variation from target population.

### Imaging protocol

All enrolled animals were imaged using a C11 transducer with a Sonosite Edge ultrasound (US) system (Fujifilm, Bothell, WA, USA) set to 8–5 MHz in B-mode. Patients were positioned in sternal, right lateral, or left lateral recumbency, depending on patient comfort and stability, with minimal to no restraint being used during image acquisition. Sequence of sites scanned was varied based on patient's initial positioning. The following scan points were imaged in most patients based on previously established protocols: bilateral pericardial sites (PCS), bilateral chest tube sites (CTS), diaphragmatic-hepatic view (DH), cystocolic (CC) view, spleno-renal (SR) view, hepato-renal (HR) view, and hepato-renal 5th (HR5) view (6).

Focal point, gain, and depth were adjusted based on operator discretion and patient size to obtain appropriate ultrasonographic image quality, with depth being reduced at chest tube sites to improve evaluation of glide sign. Scan depth ranged from 5.1 to 8.2 cm for the CTS site and ranged from 5.1 to 10 cm for all other sites. At each scan point, two 15-s clips were collected: the first with the probe oriented in the sagittal plane, followed by the second in the transverse plane. All designated scan sites were imaged in patients to obtain baseline images, even if the sites were negative for the targeted pathology. For client owned patients, images were only obtained when client consent had been secured and when acquisition did not interfere with the administration of standard of care or compromise patient stability. Patient stabilization measures included but were not limited to fluid resuscitation, blood product administration, administration of analgesics such opioids for analgesia, and nasal or flow by oxygen therapy. No client owned patients were sedated for the purposes of image collection.

### Image processing

All US video recordings were exported from the US machine producing MP4 videos with a frame rate of 30 frames per second. For each scan site, frames were extracted from each video and sorted by injury status. Each extracted frame contained a border with US user interface information in a fixed position that contained icons and text that indicated subject information and scan depth. Each brightness or B-mode image frame was cropped with fixed coordinates to remove the border containing US user interface information and resized to 512 × 512 pixels. For the CTS scan site, motion or m-mode images were generated from b-mode videos by cropping a five-pixel-wide column from the center of each b-mode image. Cropped images from a 5-s time window were then concatenated sequentially to generate a single m-mode image. Multiple m-mode images were generated from each video by iterating this process forward through an entire video in single frame increments. Generated m-mode images were then resized to 1,000 × 400 pixels.

### Image labeling and data curation

On image capture when the videos were obtained, the attending clinician specified if the examination indicated abnormal fluid or air on a data collection sheet. The videos were then reviewed by a diplomate of the American College of Veterinary Emergency and Critical Care with nearly 20 years of experience in the field both performing and teaching FAST exams to identify and classify free fluid and signs of air visible in the images. A video-based labeling approach was used to label all frames within a video according to the label of the video. Expert opinion was used to determine time points at which the injury was not visible and trim videos to exclude time windows lacking injury visibility while retaining frames that had visible signs of injury. Additionally, low quality videos which did not capture sufficient detail for injury evaluation were excluded from the dataset.

The videos for all scan sites were assigned a binary label of injury positive or injury negative. Pericardial site (PCS) videos were assigned as injury positive given the presence of either pericardial effusion (PCE) or pleural effusion. For the chest tube site (CTS), videos with PTX injury were labeled as positive for injury. Furthermore, diaphragmatic-hepatic (DH) videos were labeled injury positive given the presence of peritoneal effusion. The DH dataset includes videos with both peritoneal and pleural effusion; however, videos with only pleural and no peritoneal effusion were excluded due to limited data for proper stratification.

The SR, hepato-renal umbilical (HRU), and available HR5 videos were individually reviewed for binary injury labeling. For each subject, peritoneal effusion was not necessarily visible in videos taken at every scan site. The SR, HRU, and HR5 videos were combined into a single dataset for model development given the similarity in anatomy and effusion signs at each site. This dataset was then further curated to include the videos of the site(s) with the best representation of injury status for each subject, based on video quality and given the need for proper stratification by injury status for model training and cross-validation. Finally, videos of the CC site were also assigned a binary label of injury positive or injury negative based on the determined presence of abdominal hemorrhage.

### AI model training

**Figure 1:**
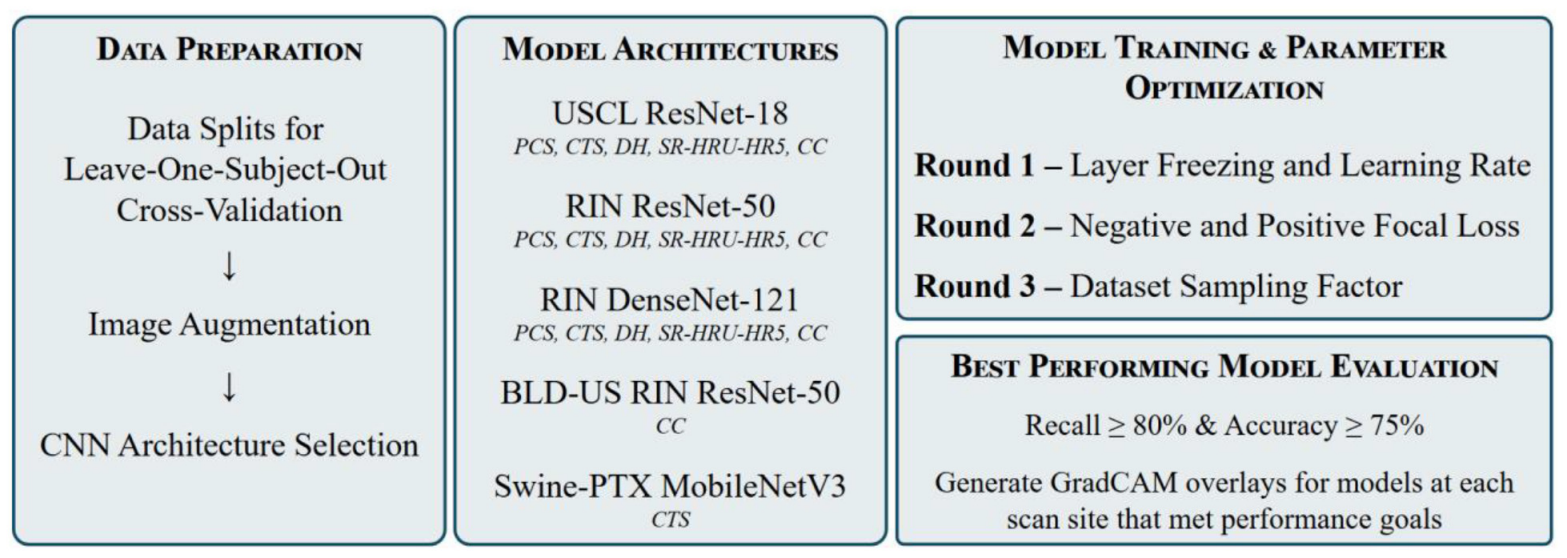
Overview of model development methodology.

An overview of the AI model training pipeline used is shown in Figure 1 from initial image acquisition through obtaining model results. This section describes (i) how data was split for cross validation, (ii) image augmentation procedures, (iii) convolutional neural network (CNN) models used, (iv) the model optimization process, and (v) how performance for trained models was assessed.

#### Leave-One-Subject-Out dataset splitting

In this study, models were developed using a Leave-One-Subject-Out (LOSO) stratified k-fold Cross-Validation (CV) approach. LOSO is a training technique that evaluates how well the model performs on unseen data from a blind subject. A model is trained by leaving one subject out of the training dataset and using the single left out subject for the test dataset, repeating this process for each subject in the dataset. Additionally, LOSO k-fold cross-validation involves leaving the one subject out and splitting the remaining training dataset into *k* number of subsets, allowing each subset an opportunity to be used as the validation set, while all other subsets are used for the training dataset. Subject-level stratification ensures that each fold of the cross-validation process maintains the same subject-level class distribution as the entire dataset. Entire subjects were assigned to folds to prevent the same subject from appearing in both the training and validation set to improve model generalization, prevent data leakage, and provide a more realistic estimate of model performance on unseen subjects.

Of the total *n* subjects in the overall dataset, *k* models were trained for each LOSO subject, resulting in a total of n^*^k number of trained models. Three k-folds were used for DH, SR-HRU-HR5, and CC model development, while two k-folds were used for PCS and CTS model development to stratify the limited number of subjects within the minority class of each site. The LOSO CV approach allowed for average model performance on blind subjects to then be evaluated using descriptive statistics.

Additionally, training and validation datasets were compiled by down sampling the total available data. A default down sampling factor of 5% was applied to the majority class. The down sampling factor of 5% was chosen to reduce the computational cost of model training and validation across LOSO splits. The down sampling factor was initially treated as a hyperparameter and iteratively reduced to a value of 5% based on training efficiency and model performance. A down sampling factor of 5% adequately maintained performance while providing training efficiency. The effect of the down sampling factor on model convergence and performance was further explored by increasing the down sampling factor and thereby increasing the dataset size during the later stages of model optimization.

Frames were sampled in sequential order to avoid redundancy from similar frames as videos captured at 30 frames per second with a US probe held in the same position rather than searching the scan sites by manually translating or tilting the probe position.

The class-balancing sampling factor required to balance the total frames in the minority class with the total frames within the majority class was then calculated for each subject and applied. Meanwhile, models were tested on 100% of the available test subject frames to allow for consistency in model performance comparison.

#### Image augmentations

Training set images were augmented by randomly applying horizontal flip (*p* = 0.5), affine transformations of 10% horizontal and vertical translation, ±10 degrees of *x*-axis shear, rotations of ±15 degrees, a scale factor of 0.8–1.2, and color brightness and contrast jitter of 0.5–1.5. All images were normalized and resized to 224 × 224. Training set images for the CTS site applied rotations of ±5 degrees and did not apply horizontal flipping or additional resizing.

#### CNN model architectures

Transfer learning is a model training approach that allows the model to pre-learn image features from a separate, large dataset for improved training performance on a new dataset for a new classification objective. Databases of medical images have been used to pre-train publicly available models that can be used for further refinement on smaller datasets for new model objectives.

AI models were developed using a transfer learning approach that leveraged open-source models pre-trained on medical images of human patients. The pre-trained models were initialized and further trained for injury diagnosis in canines in this study.

The RadImageNet (RIN) database is an open-access medical imaging database that includes 1.35 million annotated CT, MRI and ultrasound images of musculoskeletal, neurologic, oncologic, gastrointestinal, endocrine, and pulmonary pathology (28). The database includes 389,885 US images, including US images of the abdomen/pelvis and the thyroid.

ResNet-50 and Densenet-121 models pre-trained on the RIN database were used in the current study. ResNet-50 is a 50-layer CNN that uses residual learning and skip connections to enable the model to learn deeper architectures for complex image classification tasks (29). DenseNet-121 is a 121-layer CNN that uses dense connections to link each layer to all subsequent layers, enabling feature propagation and feature reuse that allows the model to learn complex image features for classification tasks (30).

Previous attempts at identifying hemorrhage in US images taken at the CC scan site have shown that identification of free fluid around the bladder, which contains fluid itself, may be a complex task. It was theorized that adapting the pre-training dataset to be even more relevant to the current task by limiting the dataset to only bladder-relevant US images would improve model performance on the current task. Therefore, an additional ResNet-50 model was pretrained using only US images available in the RIN dataset that were labeled for classification of the bladder, uterus, and ovaries.

The resulting BLD-US RIN sub-dataset was comprised of 17,665 images. Of the total dataset, 60% of the images were assigned to the training set, 30% to the validation set, and 10% to the test set. A 3-class ResNet-50 model was then pretrained for 100 epochs reaching training, validation, and testing accuracies of 92, 92, and 92%, respectively.

Additionally, the US contrastive learning (USCL) database is an US dataset that contains over 23,000 images from the Liver Fibrosis, COVID-19 LUSMS, Butterfly, and CLUST datasets for identifying liver, lung, and breast tissue pathologies (31–33). An open-source ResNet-18 model, an 18-layer CNN pretrained using the USCL dataset, was used in the current study.

Three model architectures (USCL ResNet-18, RIN ResNet-50, and RIN DenseNet-121) were trained and evaluated for all scan sites. The three models were developed by initializing existing weights pre-trained using the respective RIN and USCL medical imaging datasets and then further training the models on the canine datasets. A model for the CC site was additionally trained with the ResNet-50 model pre-trained with the BLD-US RIN sub-dataset. Finally, the CTS scan site was additionally trained with a MobileNetV3 model pretrained for a similar task of identifying PTX in POCUS images of swine with 85% blind test accuracy (34).

#### Training parameters

Models were trained by initializing the backbone architecture, loading the respective pre-trained model weights, and adapting the classifier head with a new dropout (*p* = 0.5) and linear layer for a binary classification task. Training was performed using PyTorch (Python 3.10, PyTorch 2.2+cu121, Python Software Foundation, Beaverton, OR, United States).

Models were trained with a batch size of 32 images using the Adam optimizer for up to 100 epochs. Early stopping was applied if there was no improvement to the validation loss after 5 epochs. An initial learning rate (LR) of either 1E-03 or 1E-05 was assigned with a cosine annealing learning rate scheduler. To address class imbalances, models were trained by assigning either cross-entropy loss using sample-based class weights, a positive focal loss (alpha = 0.75, gamma = 2.0) function, or a negative focal loss (alpha = 0.25, gamma = 2.0) function.

#### Performance evaluation

Several metrics were used to evaluate model prediction performance on blind subject tests. Confusion matrices were constructed by comparing model predictions to ground-truth labels. Confusion matrices for each blind test consisted of summed cases of True Positive (TP), True Negative (TN), False Positive (FP), and False Negative (FN) outcomes. These outcomes were then used to calculate accuracy, precision, recall (sensitivity), specificity, and F1-scores. Average performance metrics for each model were calculated by averaging metrics across cross-validation folds.

Additionally, recall, the True Positive Rate (TPR), was plotted against the False Positive Rate (FPR), calculated as 1 – specificity. The resulting receiver operating characteristic (ROC) curves demonstrated the diagnostic performance of the model across varying confidence thresholds. The area under the ROC curve (AUROC) was calculated as an indicator of the model's ability to distinguish between positive and negative injury states.

#### Model optimization

Recall and accuracy metrics were used during model optimization to target improved performance. A recall of 80% or greater was prioritized during model training and development so that models were able to accurately predict positive instances of injury on blind subject tests for further review by the end user. An accuracy of 75% or greater was used as a secondary metric threshold to prioritize model development toward generally robust models that were able to differentiate positive and negative injury cases, even in the case of imbalanced classes which may have skewed individual performance metrics.

Model performance was iteratively improved by varying training parameters. For each architecture, the model was initialized with the relevant pre-trained weights, and the model was trained with and without freezing all feature extraction layers of the backbone architecture. Freezing the backbone prevents the model layer weights from being updated during training for transfer learning, while unfreezing allows them to be updated for fine-tuning. Freezing the layers leverages pre-trained model weights that have captured image features, while unfreezing layers further refines the pretrained model weights for a new, specific task. Two LRs of 1E-03 and 1E-05 were applied to both training states with frozen and unfrozen layers. For all four training scenarios, the cross-entropy loss function utilized sample-based class balanced weighting. Model performance was evaluated on all four model training combinations.

Of the four training scenarios, the scenario with the highest recall and accuracy was then selected for further optimization in a second round of training. In this second round of optimization, a positive or negative focal loss was applied during training and validation.

Of the three training scenarios applying balanced weighting, negative focal loss, and positive focal loss, the best performing model was identified and further evaluated in a third round of optimization. In this third round of training, the best performing model was trained by down sampling the majority class to 10 and 25% of the available data. In these training scenarios, the effect of increasing the size of training and validation datasets was evaluated, and a final best performing model set was selected.

For the final best performing model set for each scan site, explainability was further illustrated through gradient-weighted class activation mapping (GradCAM) (35). For each training LOSO and validation fold, heat map overlays for 50 test predictions were generated. These heat map overlays illustrated areas of model attention that were most influential on the prediction outcome for each image. GradCAM image overlays were used to further understand model performance as highlighted areas of influence were expected to correspond with clinical signs of injury or other anatomical indicators.

## Results

### Patient demographics

Five military working dogs (MWDs) that were either undergoing orchiectomy or abdominal ultrasound were enrolled for image collection. This population included two German Shepherd Dogs and three Belgian Malinois. All MWDs imaged were intact males with ages ranging from 2 to 6 years (median age, 2 years). Weights in MWDs ranged from 30 to 43 kg (median weight 36 kg). None had evidence of effusion or PTX noted during image collection.

Twenty dogs were enrolled in the study, yielding a total of 21 imaging datasets. Patient age ranged from 1 to 13 years old (median 9 years). The study population included Labrador Retrievers (*n* = 5), Golden Retrievers (*n* = 4), German Shepherd Dogs (*n* = 3), mixed-breed dogs (*n* = 3), Vizsla (*n* = 1), Rhodesian Ridgeback (*n* = 1), Belgian Malinois (*n* = 1), Pitbull Terrier (*n* = 1), and Australian Shepherd (*n* = 1). Ten patients were neutered males, two were intact males, and eight were spayed females. Weights of client owner animals ranged from 23 to 39.2 kg (median weight 29.6 kg).

At presentation, four dogs were diagnosed with PTX, three with abdominal effusion, two with pleural effusion, and one with pericardial effusion. Three patients demonstrated combined pericardial and pleural effusion, two had pericardial and abdominal effusion, two had pleural and abdominal effusion, and one presented with pleural effusion in combination with PTX. Two patients exhibited tri-cavitary effusion. One dog initially diagnosed with isolated pericardial effusion developed concurrent pleural effusion the following day, prompting repeat imaging providing an additional data set. In five patients, imaging was restricted to a single body cavity due to time constraints during stabilization.

Underlying etiologies included pulmonary bullae (*n* = 1), blunt trauma (confirmed or strongly suspected; *n* = 4), cardiac neoplasia (*n* = 4), spontaneous hemoabdomen (*n* = 6), septic abdomen (*n* = 1), pyothorax (*n* = 2), and undetermined causes (*n* = 2).

In 13 of 21 imaging datasets (one patient with repeated imaging due to newly developed pleural effusion), portions of the patient's body were shaved prior to image acquisition. The timing of image collection was recorded in 18/21 datasets. The average duration required to image a single body cavity was 8.3 min (range: 5–15 min), whereas the average duration to image both cavities was 15.2 min (range: 10–20 min).

### Image datasets

A total of 283 ultrasound videos were captured for all client-owned dogs, and 131 videos were captured for MWDs for a total of 414 videos captured for all canines enrolled in the study. Of the total US video dataset, 220 were included for frame extraction and model training.

**Figure 2:**
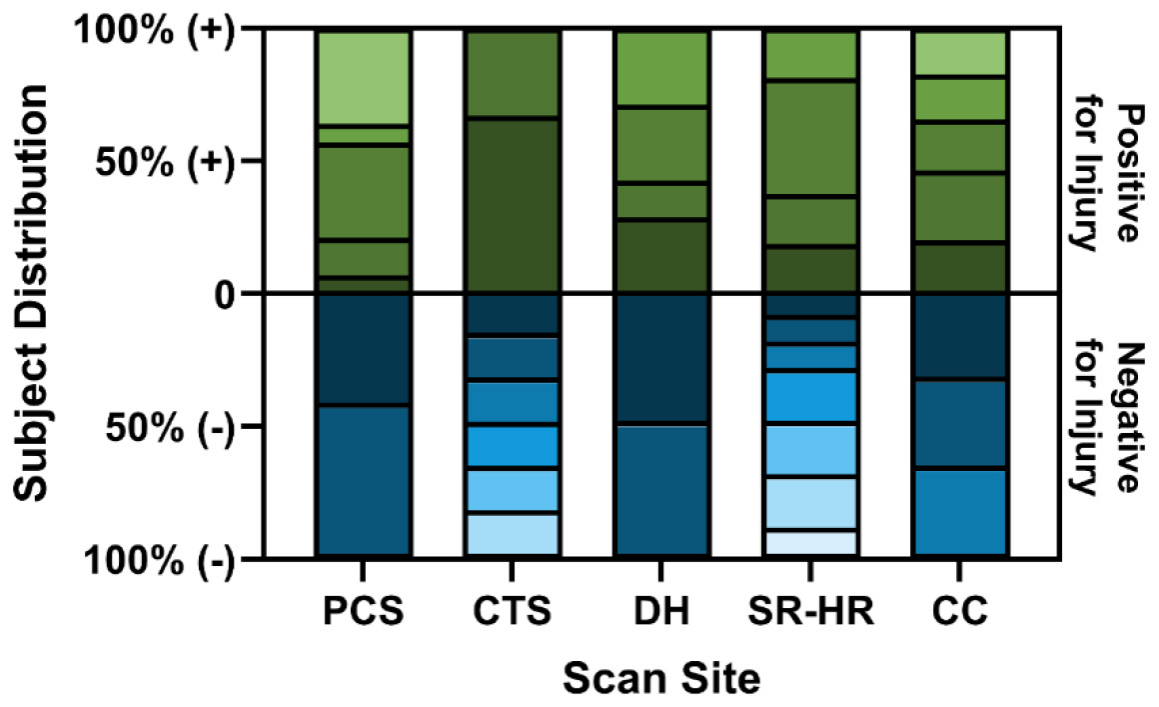
Subject-wise frame distribution per scan site demonstrating the percent distribution of frames that were used for each subject after frame sampling for each scan site for LOSO 1, Fold 1. Each color gradient represents a different subject for both positive and negative classes for each scan site.

A summary of videos and extracted frames across captured data are summarized in Table 1. Data were not evenly captured, leading to class imbalances for many of the scan sites. SR, HRU, HR5 sites were merged for the purpose of AI model development into a single model name SR-HR. For training AI models, a LOSO methodology was used and reduced frames were taken from each subject to account for subject imbalances (see Materials and Methods). A representative distribution plot for the first LOSO split and training fold for each model scan site is shown in Figure 2. Overall, these methods allowed for improved sampling across the dataset to ensure subject variability was accounted for during the training process.

**Table 1:** Summary of imaging dataset by number of subject and number of captured US clips.

	**PCS**	**CTS**	**DH**	**SR**	**HRU**	**HR5**	**CC**
Injury positive subjects	12	3	7	3	5	2	9
Injury negative subjects	3	14	4	9	9	0	4
Total subjects	15	17	11	12	14	2	13
Injury positive videos	46	8	13	3	9	4	18
Injury negative videos	11	58	7	18	17	0	8
Total videos	57	66	20	21	26	4	26
Injury positive frames	20,700	488	5,850	828	4,050	1,800	9,184
Injury negative frames	4,950	3,538	3,150	8,100	7,650	0	3,600
Total frames	25,650	4,026	9,000	8,928	11,700	1,800	12,784

Data are split by scan site and positive or negative injury state to showcase class balances for each scan site.

### Model performance evaluation

For each scan site, the effects of varied model architectures, layer freezing and learning rate LR parameters, loss functions, and dataset size on model performance were compared, and the best performing model set for each site was identified. Heat map tables comparing recall and accuracy for the varied architectures, layer states, and learning rates were generated for each scan site. The effect of further optimization testing on accuracy and recall through varied loss functions was also plotted. Additionally, the final round of optimization performance through dataset size increases is shown. Finally, the overall best performing model set's training, validation, and testing performance metrics (accuracy, precision, recall, specificity, and F1 score) were compared. Confusion matrices were normalized to represent the percentage of TP, FP, FN, and TN predictions of the total prediction outcomes and used to calculate other performance metrics.

### Pericardial site (PCS) site

**Figure 3:**
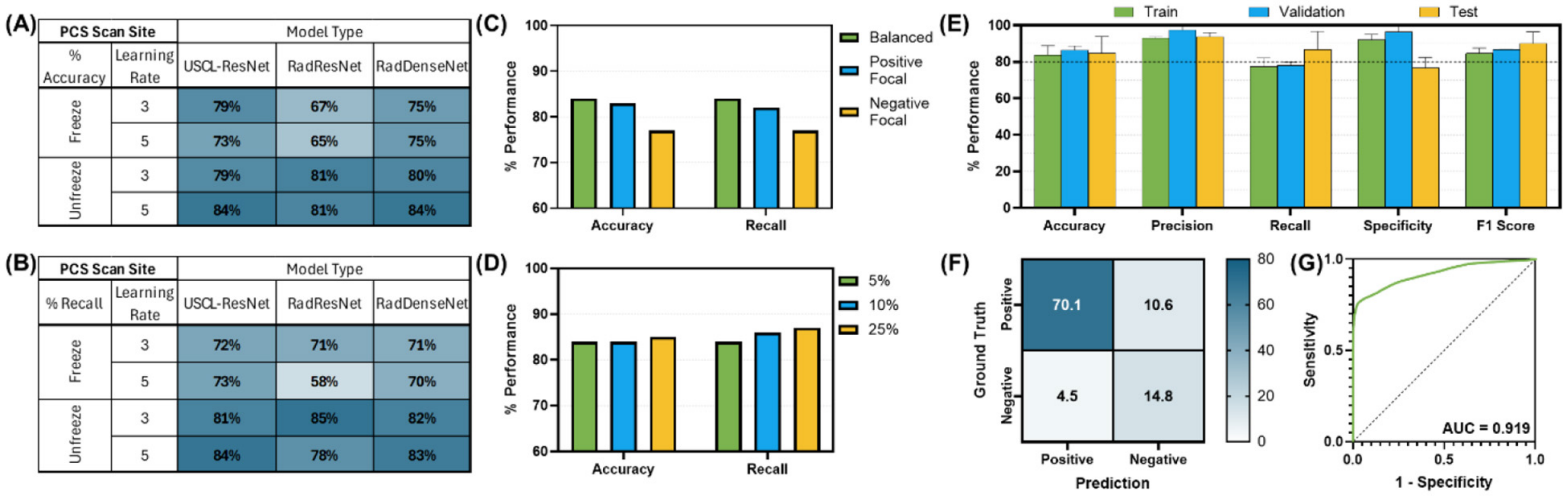
AI model optimization and performance results for PCS site. AI model optimization testing results for **(A)** accuracy, **(B)** recall, **(C)** different focal loss types, and **(D)** different percentage of training data sampling factor used. **(E)** Training, validation, and test performance metrics, **(F)** confusion matrix for blind test results, and **(G)** receiver operating characteristic curve are shown for optimal AI model configuration. Error bars denote standard deviation.

**Figure 4:**
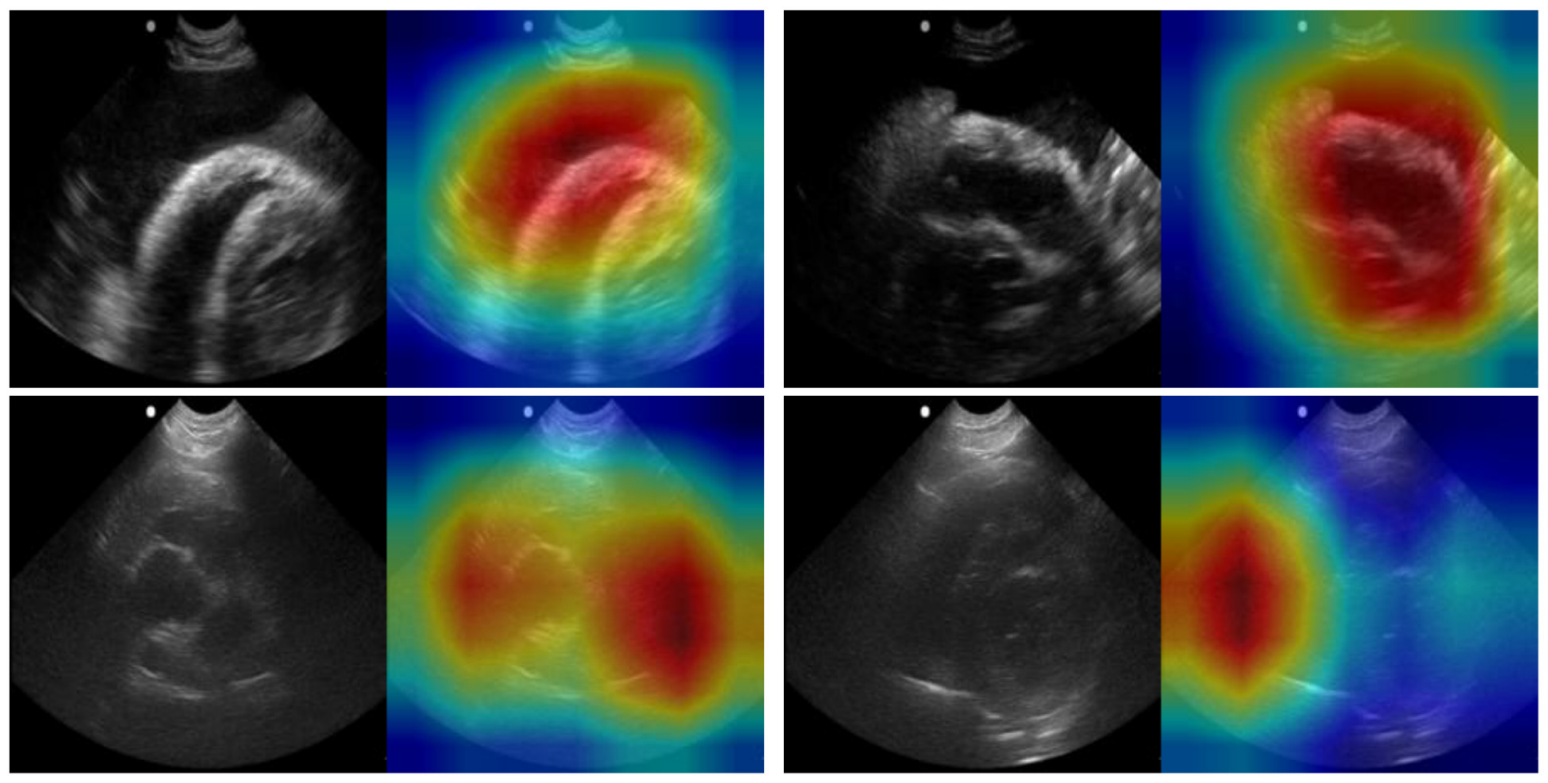
GradCAM overlays for representative predictions for PCS site. Accurate positive **(top row)** and negative **(bottom row)** predictions are shown with and without GradCAM overlay.

Multiples models were made for the detection of both pleural and pericardial effusion. Each model architecture performed similarly, with passing models for each architecture (Figure 3); however, the best performance was observed for the USCL-ResNet architecture (Unfreeze Layers, LR 1E-03). The overall best performance was achieved with a balanced weighted loss using a majority class sampling factor of 25%. For this model, average test recall and accuracy across folds were 87 ± 10% and 85 ± 9%, respectively, for the detection of pericardial or pleural effusion at the PCS site. The normalized confusion matrix had an average of 15 ± 1% TN, 5 ± 1% FP, 11 ± 8% FN, and 70 ± 8% TP outcomes. An AUROC of 0.92 was calculated from the ROC curve. GradCAM images for example PCS model predictions are shown in Figure 4 and highlighted how the AI model prediction generally correlated with image regions where the injury was noticeable.

### Chest tube site (CTS) site

**Figure 5:**
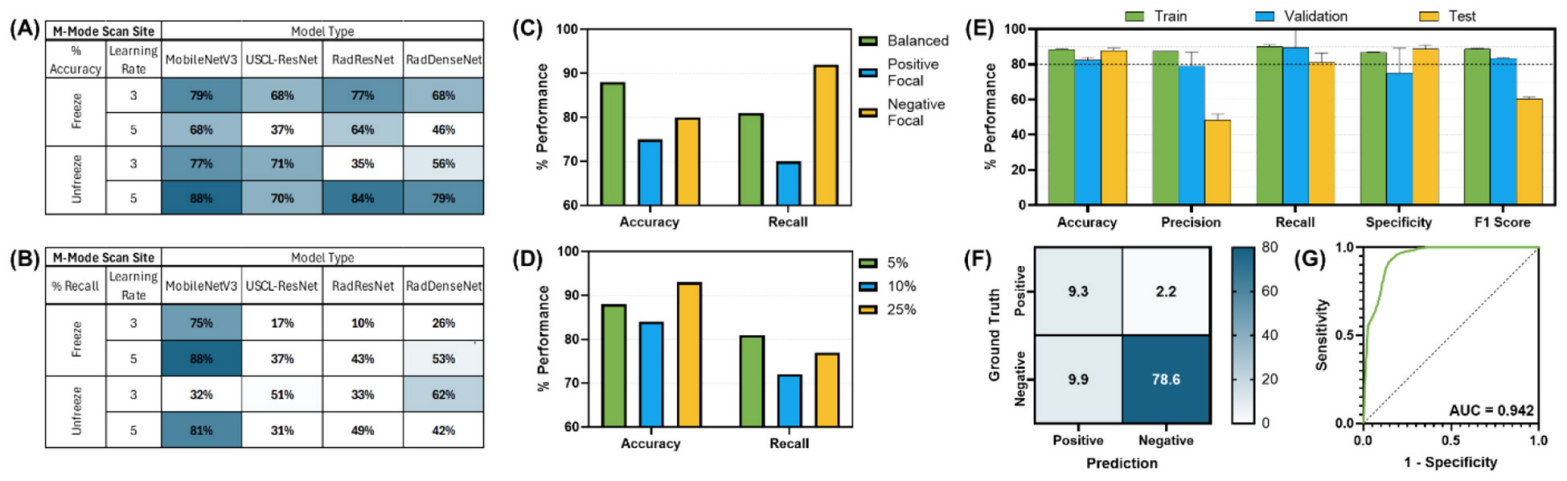
AI model optimization and performance results for CTS site. AI model optimization testing results for **(A)** accuracy, **(B)** recall, **(C)** different focal loss types, and **(D)** different percentage of training data used. **(E)** Training, validation, and test performance metrics, **(F)** confusion matrix for blind test results, and **(G)** receiver operating characteristic curve are shown for optimal AI model configuration. Error bars denote standard deviation.

**Figure 6:**
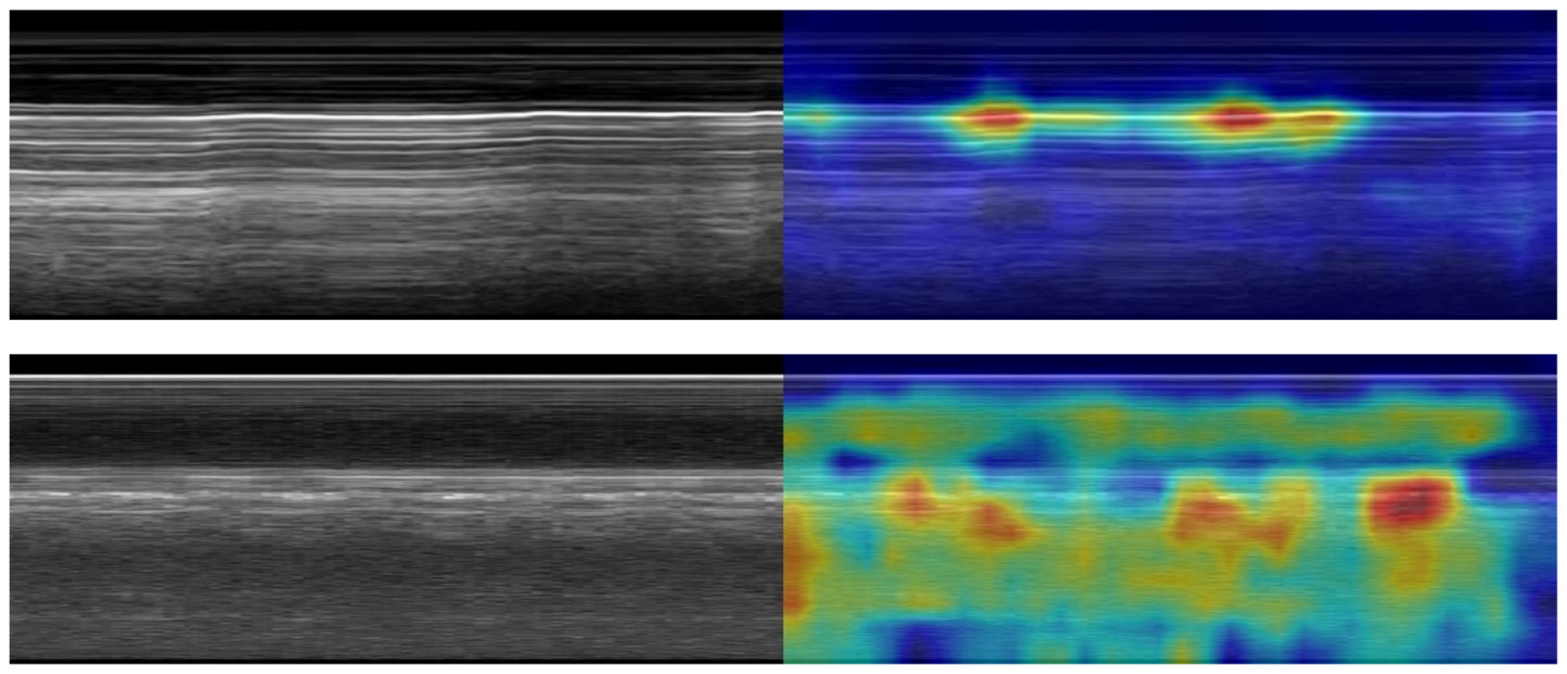
GradCAM overlays for representative predictions for CTS site. Accurate positive **(top row)** and negative **(bottom row)** predictions are shown with and without GradCAM overlay.

Due to lack of cases of pleural effusion notable at the CTS on the obtained dataset, models were only developed for the detection of PTX. The USCL-ResNet, RIN ResNet-50, and RIN DenseNet-121 models did not meet the dual criteria for recall and accuracy performance in any of the training scenarios that were evaluated (Figure 5). Recall did not surpass 62%, and few models met the accuracy threshold of 75%. However, the MobileNetV3 architecture (Unfreeze Layers, LR 1E-05) had the highest performance of the varied architectures that were trained. Of the MobileNetV3 models that were trained, a balanced weighted loss and majority class sampling factor of 5% provided the greatest model improvements. For the best performing MobileNetV3 model, blind test performance demonstrated a recall of 81 ± 6% and accuracy of 88 ± 1%. The confusion matrix demonstrated an average of 79 ± 2% TN, 10 ± 2% FP, 2 ± 1% FN, and 9 ± 1% TP prediction outcomes. An AUROC of 0.94 was calculated for the CTS site. GradCAM overlays demonstrated AI model identification of breathing artifacts or the lack of breathing in the case of PTX (Figure 6).

### Diaphragmatic-hepatic (DH) site

**Figure 7:**
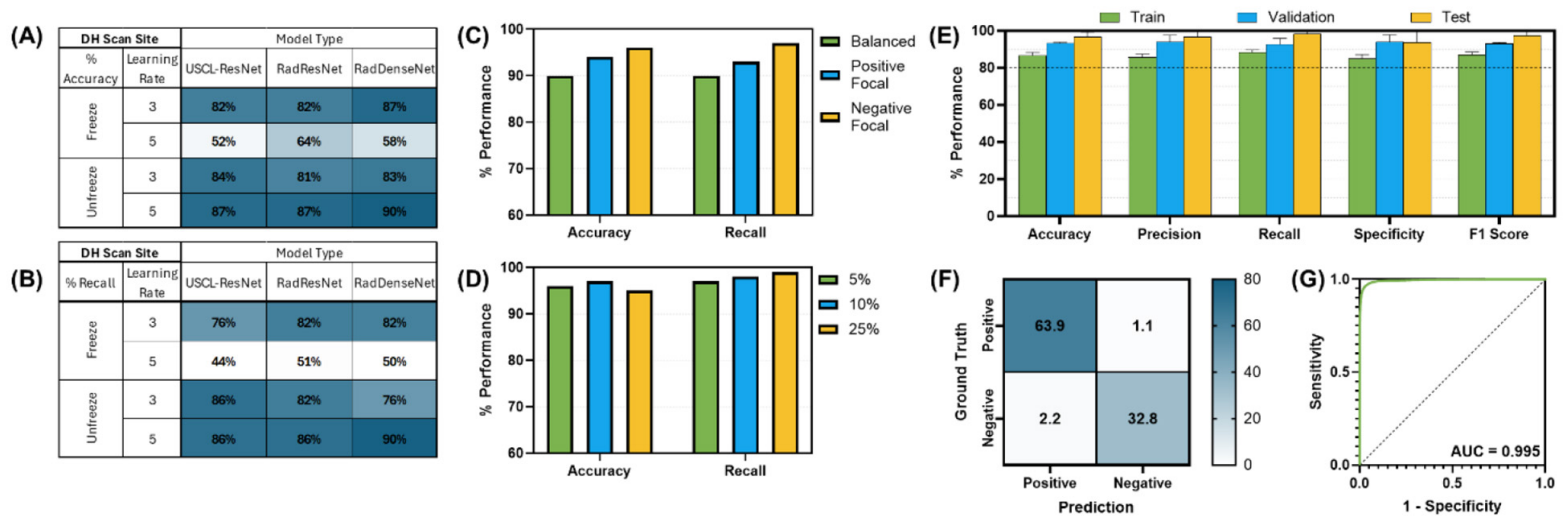
AI model optimization and performance results for DH site. AI model optimization testing results for **(A)** accuracy, **(B)** recall, **(C)** different focal loss types, and **(D)** different percentage of training data used. **(E)** Training, validation, and test performance metrics, **(F)** confusion matrix for blind test results, and **(G)** receiver operating characteristic curve are shown for optimal AI model configuration. Error bars denote standard deviation.

**Figure 8:**
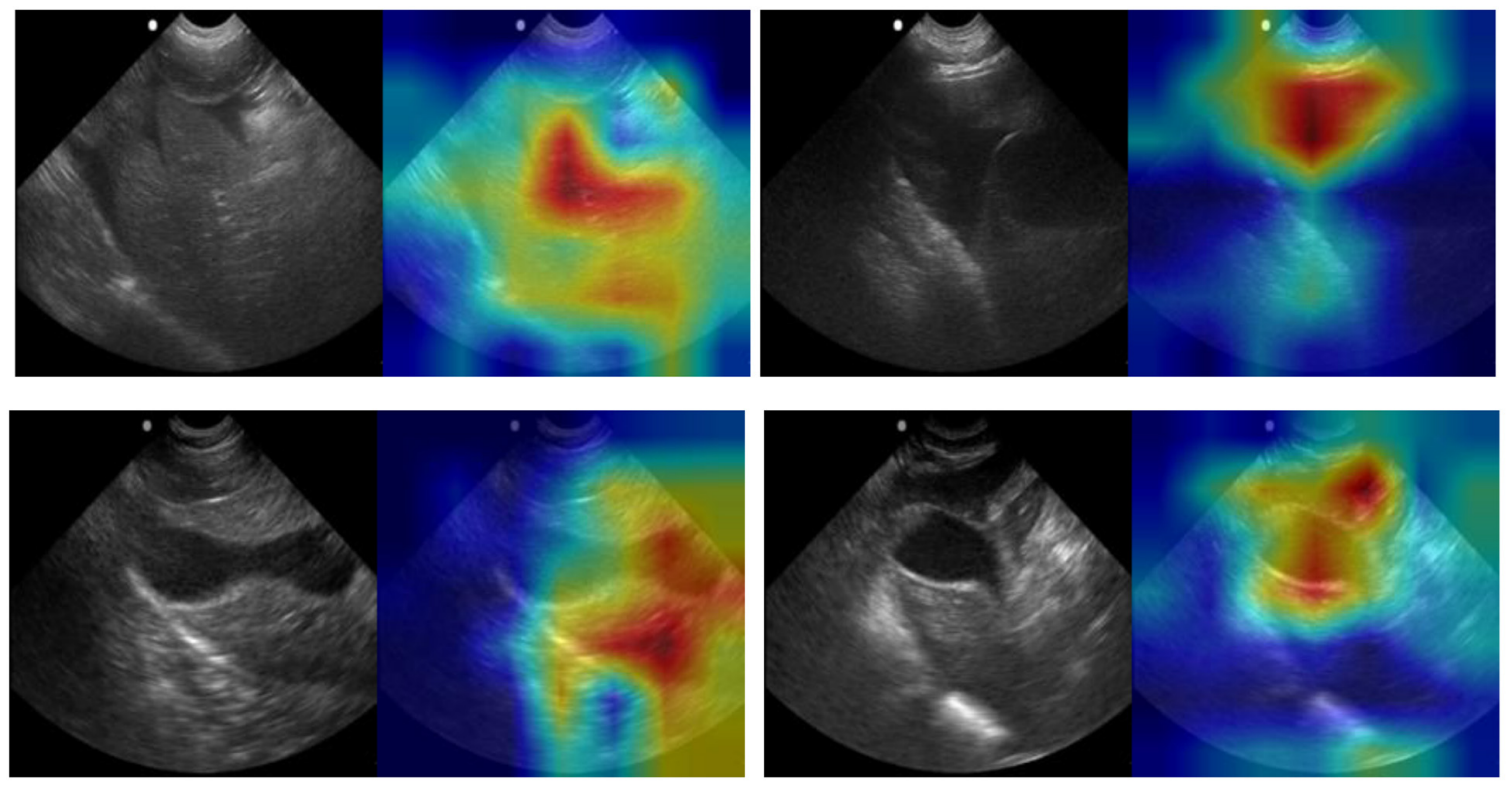
GradCAM overlays for representative predictions for DH site. Accurate positive **(top row)** and negative **(bottom row)** predictions are shown with and without GradCAM overlay.

Of the model architectures evaluated for detection of peritoneal effusion, RIN DenseNet-121 (Unfreeze Layers, LR 1E-05) achieved the best performance, with similar passing performance observed for the RIN ResNet-50 and USCL-ResNet-18 architectures (Figure 7). A negative focal loss and majority class sampling factor of 10% improved RIN DenseNet-121 model test performance to 98 ± 2% recall and 97 ± 3% accuracy for the overall best model performance. The confusion matrix demonstrated 33 ± 3% TN, 2 ± 3% FP, 1 ± 1% FN, and 64 ± 1% TP prediction outcomes. The AUROC was calculated to be 0.995. GradCAM representative overlays highlighted how the AI model was tracking key anatomical features when there was no fluid present and focused on signs of fluid for positive injury frames (Figure 8).

### Splenorenal-hepatorenal (SR-HRU-HR5) site

**Figure 9:**
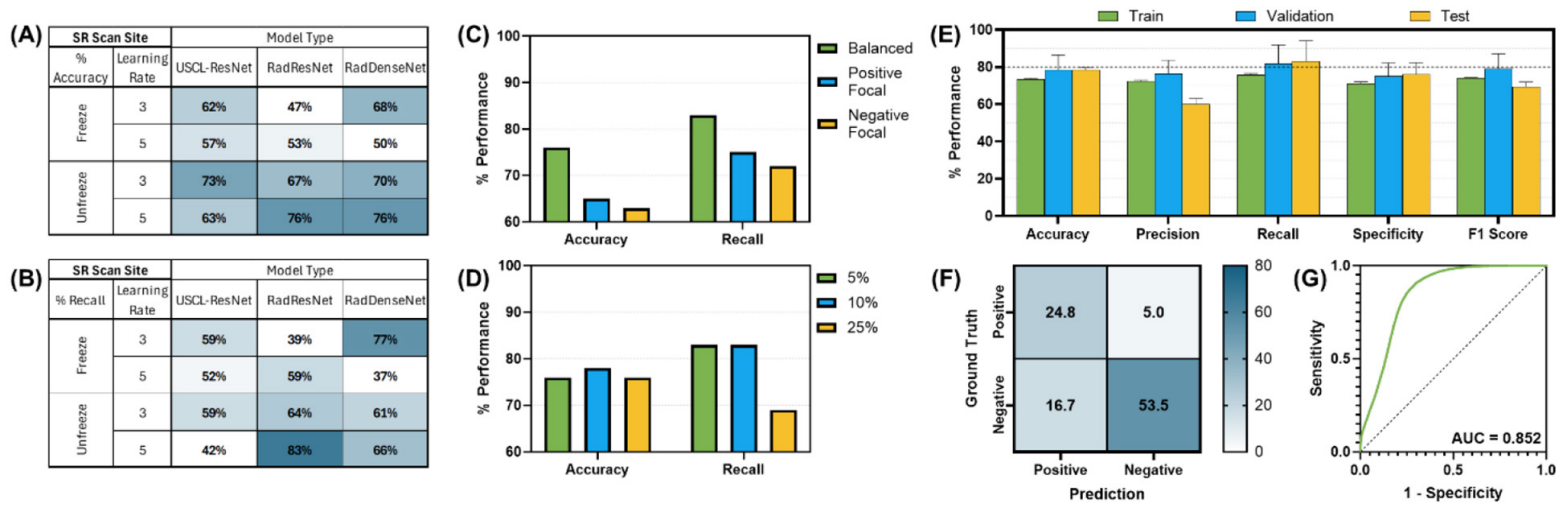
AI model optimization and performance results for SH-HRU-HR5 site. AI model optimization testing results for **(A)** accuracy, **(B)** recall, **(C)** different focal loss types, and **(D)** different percentage of training data used. **(E)** Training, validation, and test performance metrics, **(F)** confusion matrix for blind test results, and **(G)** receiver operating characteristic curve are shown for optimal AI model configuration. Error bars denote standard deviation.

**Figure 10:**
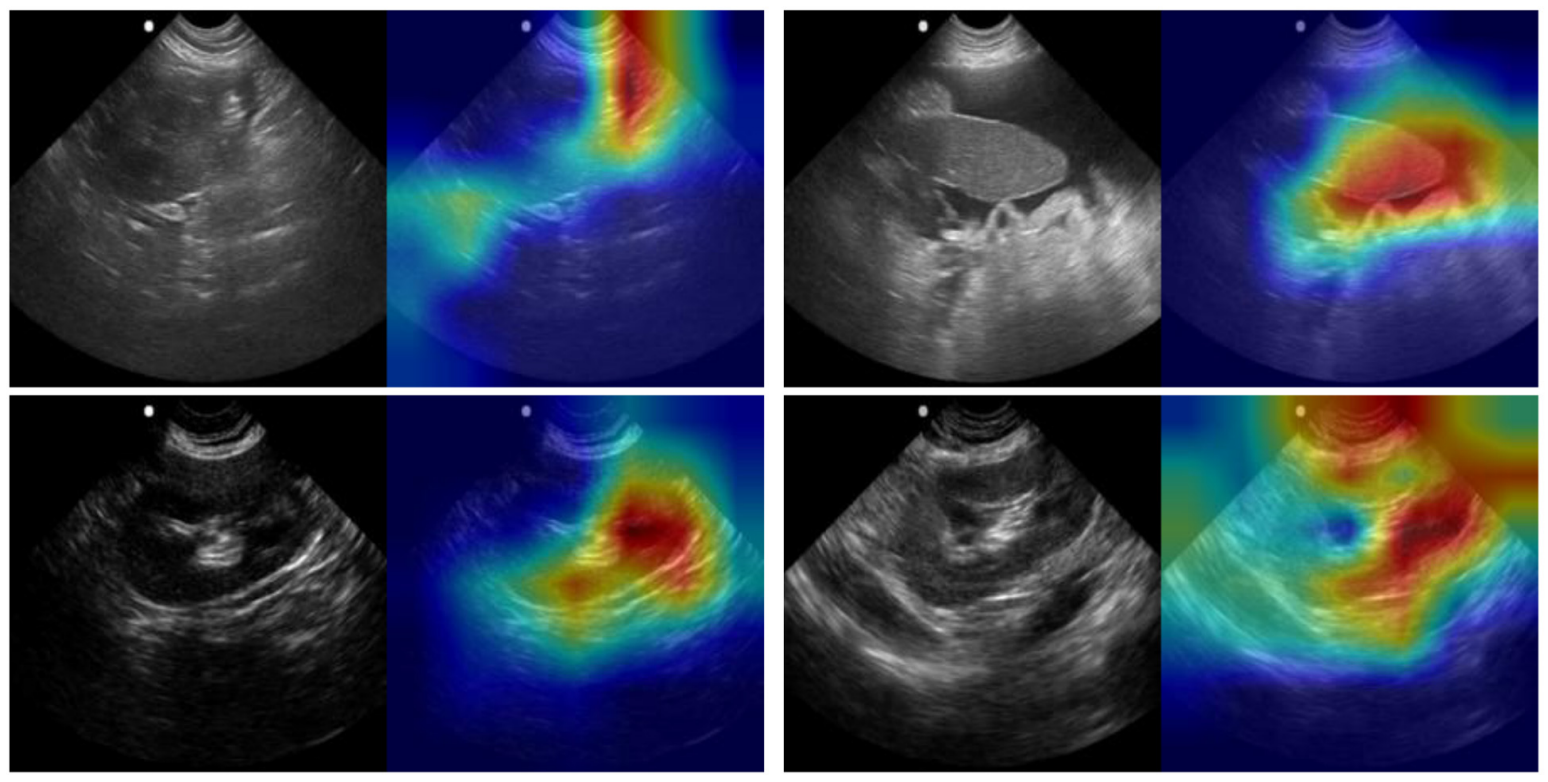
GradCAM overlays for representative predictions for SR-HRU-HR5 site. Accurate positive **(top row)** and negative **(bottom row)** predictions are shown with and without GradCAM overlay.

The best performing architecture for detection of peritoneal effusion was the RIN ResNet-50 model (Unfreeze, LR 1E-05) with passing performance, while the USCL-ResNet-18 and RIN DenseNet-121 models did not achieve a passing score, based on the dual recall and accuracy performance criteria (Figure 9). The RIN ResNet-50 model was further optimized to a recall of 83 ± 11% and accuracy of 78 ± 2% with a balanced weighted loss and majority class sampling factor of 10%. The confusion matrix showed prediction outcomes of 54 ± 4% TN, 17 ± 4% FP, 5 ± 3% FN, and 25 ± 3% TP. An AUROC of 0.85 was calculated for this site. Per GradCAM overlays, AI models correlated with the presence of fluid when presented in injury positive frames (Figure 10).

### Cystocolic (CC) site

**Figure 11:**
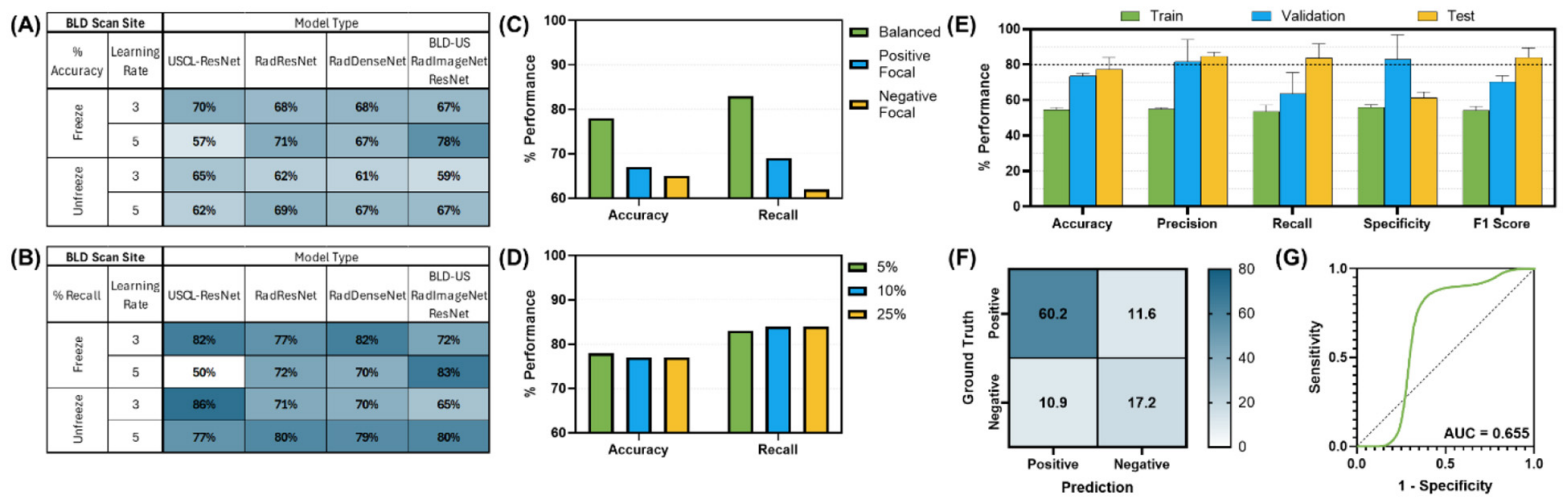
AI model optimization and performance results for CC site. AI model optimization testing results for **(A)** accuracy, **(B)** recall, **(C)** different focal loss types, and **(D)** different percentage of training data used. **(E)** Training, validation, and test performance metrics, **(F)** confusion matrix for blind test results, and **(G)** receiver operating characteristic curve are shown for optimal AI model configuration. Error bars denote standard deviation.

**Figure 12:**
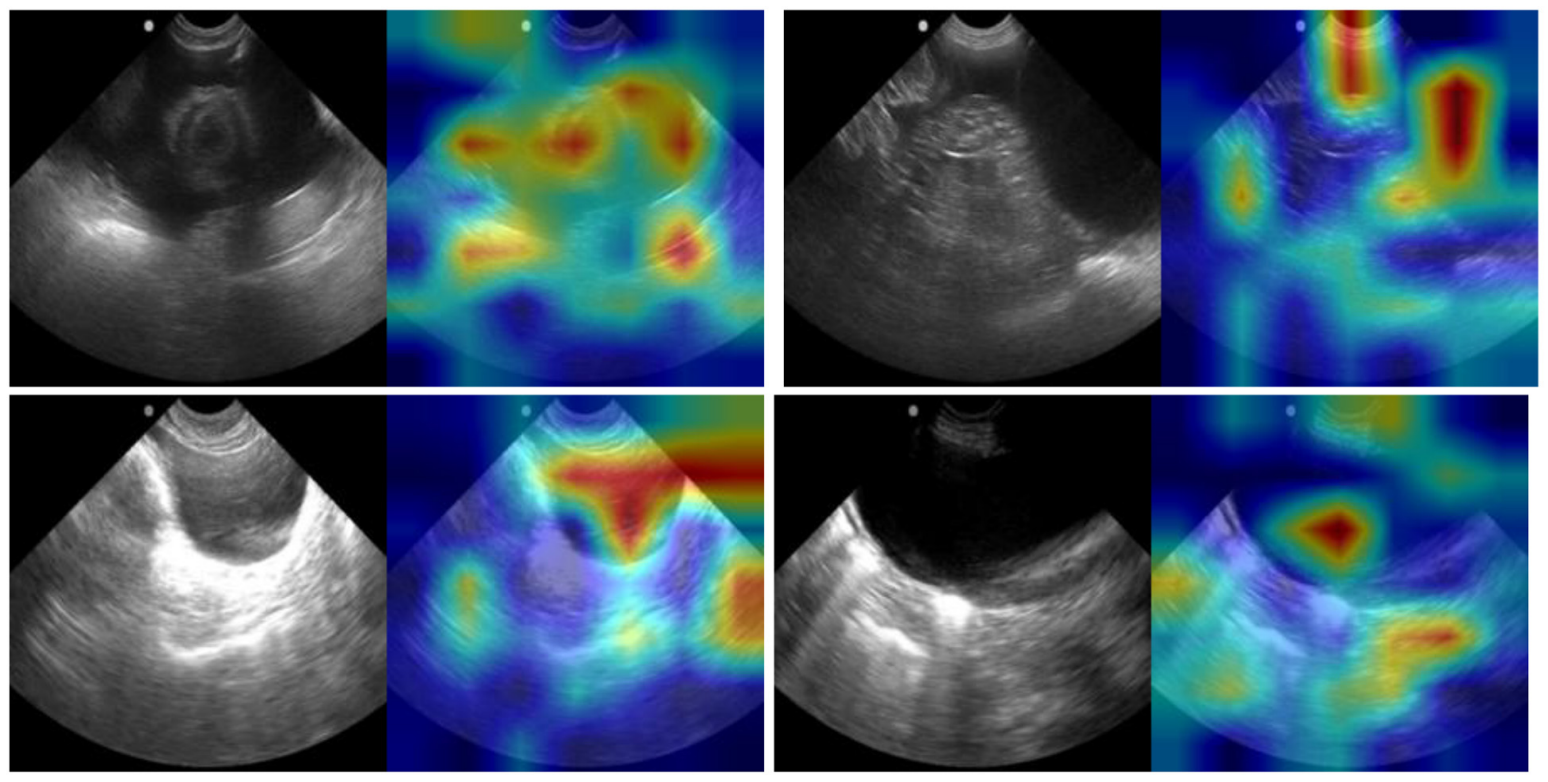
GradCAM overlays for representative predictions for CC site. Accurate positive **(top row)** and negative **(bottom row)** predictions are shown with and without GradCAM overlay.

The USCL-ResNet-18, RIN ResNet-50, and RIN DenseNet-121 model architectures did not achieve the targeted performance criteria in any of the training scenarios that were evaluated for detection of abdominal effusion (Figure 11). However, the BLD-US RIN ResNet-50 model (Freeze, LR 1E-05) pretrained with the bladder-relevant US images from the RIN dataset was able to achieve passing performance. A balanced weighted loss with a majority sampling factor of 10% further optimized blind test performance of the model with a recall of 84 ± 8% and accuracy of 77 ± 7%. The confusion matrix demonstrated 17 ± 1% TN, 11 ± 1% FP, 12 ± 6% FN, and 60 ± 6% TP prediction outcomes. An AUROC of 0.66 was calculated for the ROC curve. GradCAM overlays are shown for tracking model decision attention, improving explainability (Figure 12).

## Discussion

The best-performing models in this study were for the DH, CTS, and PCS views, demonstrating accuracies of 97, 88, and 85% and sensitivities of 98, 81, and 87%, respectively. The results of these models are comparable to diagnostics performance of trained personnel previously reported in veterinary studies (1, 2, 11–16). GradCAM overlays demonstrated improved localization of injury-associated regions with less attention to image artifacts compared with previously reported veterinary POCUS deep learning models (23). These findings suggest that, when paired with adequate image acquisition training, the algorithms could provide real-time diagnostic support to identify abnormal fluid or air. Embedding such algorithms directly into portable ultrasound units could increase diagnostic confidence for less experienced practitioners, particularly in resource-limited settings such as the battlefield or rural regions and may enable first responders to detect life-threatening pathology prior to arrival at a veterinary hospital. Importantly, on-device analysis would remove reliance on telemedicine or internet connectivity, allowing autonomous interpretation in the field.

Despite these promising results, several limitations were identified. At the PCS site, models demonstrated strong sensitivity for hemorrhage detection but reduced specificity. GradCAM analysis showed that well-performing models appropriately focused on regions of pericardial and pleural effusion and correctly interpreted shadowing as artifacts rather than assuming hemorrhage. However, false positives often arose from the difficulty of identifying scant effusion as well as distinguishing right ventricular fluid from hemorrhage. In contrast, CTS models initially performed poorly, as images in this study were highly dissimilar to those in the USCL and RIN databases. Performance improved substantially with MobileNetV3 pre-trained on swine m-mode POCUS for PTX detection. GradCAM overlays showed that the model properly assessed area under the pleural line for evidence of lung motion. While the final model achieved high accuracy, motion artifacts introduced during probe movement contributed to false negatives, underscoring the importance of stable probe placement in future data collection.

For abdominal sites, DH models achieved the highest performance overall, likely reflecting strong domain overlap between liver-focused pre-training datasets and the diagnostic task of hemorrhage detection at the DH site. These models demonstrated both high sensitivity and specificity, with GradCAM confirming appropriate attention to regions of free fluid rather than anatomical structures such as the gallbladder.

Models for the SR-HRU-HR5 and CC views demonstrated accuracies of 78 and 77% and sensitivities of 83 and 84%. While demonstrating lower accuracy due to lower specificity, potentially as a result of class imbalances, these models demonstrated high sensitivity for abdominal hemorrhage detection, improving on sensitivities reported in prior FAST studies (11, 14). SR-HRU-HR5 models showed fair overall performance, with GradCAM's showing that there was opportunity for the model to better learn spatial features relevant to hemorrhage classification such as the size or typical location of free fluid near the kidneys. Outcomes improved when pre-training included kidney-relevant images, suggesting that additional domain-targeted pre-training could enhance future results. At the CC site, sensitivity was strong when models were pre-trained on bladder-specific datasets, but specificity was reduced due to difficulty distinguishing free peritoneal fluid from contained bladder fluid. Variability in bladder size, positioning, and free-fluid appearance added diagnostic complexity and contributed to underfitting.

Limitations of this study included the use of only one US probe to capture images, the small number of subjects available in the canine dataset, inclusion of only blunt trauma and non-traumatic pathology, and presence of class imbalances, with few uninjured subjects available for PCS, CTS, DH, and CC model development. While average performance achieved targeted thresholds, models showed high variation in performance across validation folds and blind subject LOSO's. Future work should prioritize expanding dataset size, with a focus on balancing classes with larger numbers of uninjured controls and representing injury variability with more diverse presentations of effusion and air. Improvements to the training and evaluation dataset would allow models to be more robust to subject variability and improve free fluid detection for a range of injury types and severities. Improved dataset quality would support training of more sophisticated architectures and facilitate models capable of distinguishing among effusion types. Continued refinement of both datasets and model architectures will be essential for reliable integration into clinical and pre-hospital veterinary practice.

In future work, training datasets should be expanded to overcome limitations imposed by the small size of the datasets, class imbalances, and the high degree of subject variability and injury presentation observed within the current study. The algorithms developed here should also be applied to other US platforms to determine if the algorithms can be universally applied to different US machines. Additionally, models should be further developed to differentiate pericardial effusion, pleural effusion, and abdominal hemorrhage in instances of multiple types of effusion. Furthermore, model explainability would be improved through the development of image segmentation models that not only classify the injury status of the image but also highlight the boundaries of the injury. For clinical implementation, these efforts could improve injury status evaluation and user confidence in diagnostic assessments. With further model development, efforts should focus on real-time implementation of the developed models through the development of strategies that provide a wholistic diagnostic determination from US video even with motion and other scanning artifacts.

## Conclusion

Point-of-care ultrasound is an indispensable tool for veterinary triage and, if implemented in the pre-hospital setting, could improve patient outcomes. AI, as demonstrated in this study, offers a potential solution by providing real-time, accurate automated interpretation of ultrasound images when trained personnel are unavailable. Our findings show that ultrasound examinations can be performed rapidly during the time-sensitive triage process, with AI algorithms being developed to inform diagnostic processes at the point of injury which can help guide early intervention before referral to facilities with specialized expertise. Diagnostic AI models were developed to achieve 80% sensitivity and 75% accuracy or greater at the detection of PTX and pericardial and abdominal hemorrhage. Models for the DH, CTS, PCS, SR-HRU-HR5, and CC views achieved sensitivity of 98, 81, 87, 83 and 84% with accuracy of 97, 88, 85, 78, and 77%, respectively. Diagnostic AI models show significant potential for aiding medical imaging-based triage and allowing these techniques to be more widely used. With further development, diagnostic AI models can assist with the detection of thoracic and abdominal injuries for MWDs and other canines with traumatic injury so that proper treatment may be delivered in emergency situations.

## Data Availability

The data presented in this study are not publicly available because they have been collected and maintained in a government-controlled database located at the U.S. Army Institute of Surgical Research. This data can be made available through the development of a Cooperative Research and Development Agreement (CRADA) with the corresponding author.

## References

[1] Boysen SR Rozanski EA Tidwell AS Holm JL Shaw SP Rush JE. Evaluation of a focused assessment with sonography for trauma protocol to detect free abdominal fluid in dogs involved in motor vehicle accidents. J Am Vet Med Assoc. (2004) 225:1198–204. doi: 10.2460/javma.2004.225.1198 15521440

[2] Lisciandro GR Lagutchik MS Mann KA Voges AK Fosgate GT Tiller EG . Evaluation of a thoracic focused assessment with sonography for trauma (TFAST) protocol to detect pneumothorax and concurrent thoracic injury in 145 traumatized dogs. J Vet Emerg Crit Care. (2008) 18:258–69. doi: 10.1111/j.1476-4431.2008.00312.x

[3] Lisciandro GR Lagutchik MS Mann KA Fosgate GT Tiller EG Cabano NR . Evaluation of an abdominal fluid scoring system determined using abdominal focused assessment with sonography for trauma in 101 dogs with motor vehicle trauma. J Vet Emerg Crit Care. (2009) 19:426–37. doi: 10.1111/j.1476-4431.2009.00459.x 19821883

[4] Lisciandro GR. Abdominal and thoracic focused assessment with sonography for trauma, triage, and monitoring in small animals. J Vet Emerg Crit Care. (2011) 21:104–22. doi: 10.1111/j.1476-4431.2011.00626.x 21463438

[5] McMurray J Boysen S Chalhoub S. Focused assessment with sonography in nontraumatized dogs and cats in the emergency and critical care setting. J Vet Emerg Crit Care. (2016) 26:64–73. doi: 10.1111/vec.12376 26445109

[6] Lisciandro GR. Cageside ultrasonography in the emergency room and intensive care unit. Vet Clin North Am Small Anim Pract. (2020) 50:1445–67. doi: 10.1016/j.cvsm.2020.07.013 32912606

[7] Storer AP Edwards TH Rutter CR Young GE Mullaney SB. Causes of mortality in military working dog from traumatic injuries. Front Vet Sci. (2024) 11:1360233. doi: 10.3389/fvets.2024.1360233 39040817 PMC11260784

[8] Parlak K Zamirbekova N Uzunlu EO Akyol ET Yavru N. Comparison of the focused assessment with sonography for trauma protocol and animal trauma triage scoring system in traumatized dogs. Kafkas Üniversitesi Veteriner Fakültesi Dergisi. (2021) 27:439–44. doi: 10.9775/kvfd.2021.25457

[9] Dicker SA. Lung ultrasound for pulmonary contusions. Vet Clin North Am Small Anim Pract. (2021) 51:1141–51. doi: 10.1016/j.cvsm.2021.07.001 34521570

[10] Pelchat J Chalhoub S Boysen SR. The use of veterinary point-of-care ultrasound by veterinarians: a nationwide Canadian survey. Can Vet J. (2020) 61:1278–82. 33299243 PMC7659883

[11] Netherton S Milenkovic V Taylor M Davis PJ. Diagnostic accuracy of eFAST in the trauma patient: a systematic review and meta-analysis. CJEM. (2019) 21:727–38. doi: 10.1017/cem.2019.381 31317856

[12] Staub LJ Biscaro RRM Kaszubowski E Maurici R. Chest ultrasonography for the emergency diagnosis of traumatic pneumothorax and haemothorax: a systematic review and meta-analysis. Injury. (2018) 49:457–66. doi: 10.1016/j.injury.2018.01.033 29433802

[13] Partyka C Coggins A Bliss J Burns B Fiorentino M Goorkiz P . multicenter evaluation of the accuracy of prehospital eFAST by a physician-staffed helicopter emergency medical service. Emerg Radiol. (2022) 29:299–306. doi: 10.1007/s10140-021-02002-4 34817706

[14] Smith IM Naumann DN Marsden MER Ballard M Bowley DM. Scanning and war: utility of FAST and CT in the assessment of battlefield abdominal trauma. Ann Surg. (2015) 262:389–96. doi: 10.1097/SLA.0000000000001002 25405557

[15] Walters AM O'Brien MA Selmic LE Hartman S McMichael M O'Brien RT. Evaluation of the agreement between focused assessment with sonography for trauma (AFAST/TFAST) and computed tomography in dogs and cats with recent trauma. J Vet Emerg Crit Care. (2018) 28:429–35. doi: 10.1111/vec.12732 29901282

[16] Armenise A Boysen RS Rudloff E Neri L Spattini G Storti E. Veterinary-focused assessment with sonography for trauma-airway, breathing, circulation, disability and exposure: a prospective observational study in 64 canine trauma patients. J Small Animal Pract. (2019) 60:173–82. doi: 10.1111/jsap.12968 30549049

[17] Hennessey E DiFazio M Hennessey R Cassel N. Artificial intelligence in veterinary diagnostic imaging: a literature review. Vet Radiol Ultrasound. (2022) 63 Suppl 1:851–70. doi: 10.1111/vru.13163 36468206

[18] Kim E Fischetti AJ Sreetharan P Weltman JG Fox PR. Comparison of artificial intelligence to the veterinary radiologist's diagnosis of canine cardiogenic pulmonary edema. Vet Radiol Ultrasound. (2022) 63:292–7. doi: 10.1111/vru.13062 35048445

[19] Pereira AI Franco-Gonçalo P Leite P Ribeiro A Alves-Pimenta MS Colaço B . Artificial intelligence in veterinary imaging: an overview. Vet Sci. (2023) 10:320. doi: 10.3390/vetsci10050320 37235403 PMC10223052

[20] Ndiaye YS Cramton P Chernev C Ockenfels A Schwarz T. Comparison of radiological interpretation made by veterinary radiologists and state-of-the-art commercial AI software for canine and feline radiographic studies. Front Vet Sci. (2025) 12:1502790. doi: 10.3389/fvets.2025.1502790 40061904 PMC11886591

[21] Jourdan A Dania C Cambournac M. Sonographic machine-assisted recognition and tracking of B-lines in dogs: the SMARTDOG study. Front Vet Sci. (2025) 12:1647547. doi: 10.3389/fvets.2025.1647547 40860925 PMC12371216

[22] Boissady E de La Comble A Zhu X Hespel A-M. Artificial intelligence evaluating primary thoracic lesions has an overall lower error rate compared to veterinarians or veterinarians in conjunction with the artificial intelligence. Vet Radiol Ultrasound. (2020) 61:619–27. doi: 10.1111/vru.12912 32996208

[23] Hernandez Torres SI Holland L Edwards TH Venn EC Snider EJ. Deep learning models for interpretation of point of care ultrasound in military working dogs. Front Vet Sci. (2024) 11:1374890. doi: 10.3389/fvets.2024.1374890 38903685 PMC11187302

[24] Montgomery S Li F Funk C Peethumangsin E Morris M Anderson JT . Detection of pneumothorax on ultrasound using artificial intelligence. J Trauma Acute Care Surg. (2023) 94:379–84. doi: 10.1097/TA.0000000000003845 36730087

[25] Huang L Lin Y Cao P Zou X Qin Q Lin Z . Automated detection and segmentation of pleural effusion on ultrasound images using an attention U-net. J Appl Clin Med Physics. (2024) 25:e14231. doi: 10.1002/acm2.14231 38088928 PMC10795456

[26] Kim S Fischetti C Guy M Hsu E Fox J Young SD. Artificial intelligence (AI) applications for point of care ultrasound (POCUS) in low-resource settings: a scoping review. Diagnostics. (2024) 14:1669. doi: 10.3390/diagnostics14151669 39125545 PMC11312308

[27] Seyed Bolouri SE Dehghan M Nekoui M Buchanan B Jaremko JL Zonoobi D . Enhances lung ultrasound interpretation across clinicians with varying expertise levels. Diagnostics. (2025) 15:2145. doi: 10.3390/diagnostics15172145 40941633 PMC12428092

[28] Mei X Liu Z Robson PM Marinelli B Huang M Doshi A . RadImageNet: an open radiologic deep learning research dataset for effective transfer learning. Radiol Artif Intell. (2022) 4:e210315. doi: 10.1148/ryai.210315 36204533 PMC9530758

[29] He K Zhang X Ren S Sun J. Deep residual learning for image recognition. In: Proceedings of the IEEE Conference on Computer Vision and Pattern Recognition. New York, NY: IEEE (2016). p. 770–8. doi: 10.1109/CVPR.2016.90

[30] Huang G Liu Z Van Der Maaten L Weinberger KQ. Densely connected convolutional networks. In: Proceedings of the IEEE Conference on Computer Vision and Pattern Recognition. New York, NY: IEEE (2017). p. 4700–8. doi: 10.1109/CVPR.2017.243

[31] Chen Y Zhang C Liu L Feng C Dong C Luo Y . USCL: pretraining deep ultrasound image diagnosis model through video contrastive representation learning. In: International Conference on Medical Image Computing and Computer-Assisted Intervention. Cham: Springer. (2021). p. 627–37. doi: 10.1007/978-3-030-87237-3_60

[32] Born J Wiedemann N Cossio M Buhre C Brändle G Leidermann K . Accelerating detection of lung pathologies with explainable ultrasound image analysis. Appl Sci. (2021) 11:672. doi: 10.3390/app11020672

[33] Somphone O Allaire S Mory B Dufour C. Live feature tracking in ultrasound liver sequences with sparse demons. In: Proceedings of MICCAI Workshop on Challenge on Liver Ultrasound Tracking. Cambridge, MA: Medical Image Computing and Computer Assisted Interventions. (2014). p. 53

[34] Ruiz AJ Hernández Torres SI Snider EJ. Development of deep learning models for real-time thoracic ultrasound image interpretation. J Imaging. (2025) 11:222. doi: 10.3390/jimaging11070222 40710609 PMC12295020

[35] Selvaraju RR Cogswell M Das A Vedantam R Parikh D Batra D. Grad-cam: visual explanations from deep networks via gradient-based localization. In: Proceedings of the IEEE international conference on computer vision. Venice: IEEE (2017). p. 618–26. doi: 10.1109/ICCV.2017.74

